# Supplementary material for: The Antidepressant Activity of a Taurine-Containing Derivative of 4-Phenylpyrrolidone-2 in a Model of Chronic Unpredictable Mild Stress
Source: Int J Mol Sci. 2023 Nov 21;24(23):16564. doi: 10.3390/ijms242316564 (PMC10705968; doi:10.3390/ijms242316564)
Supplement: Supplementary file 1 [file ijms-24-16564-s001.zip › ijms-2703854-supplementary.pdf]

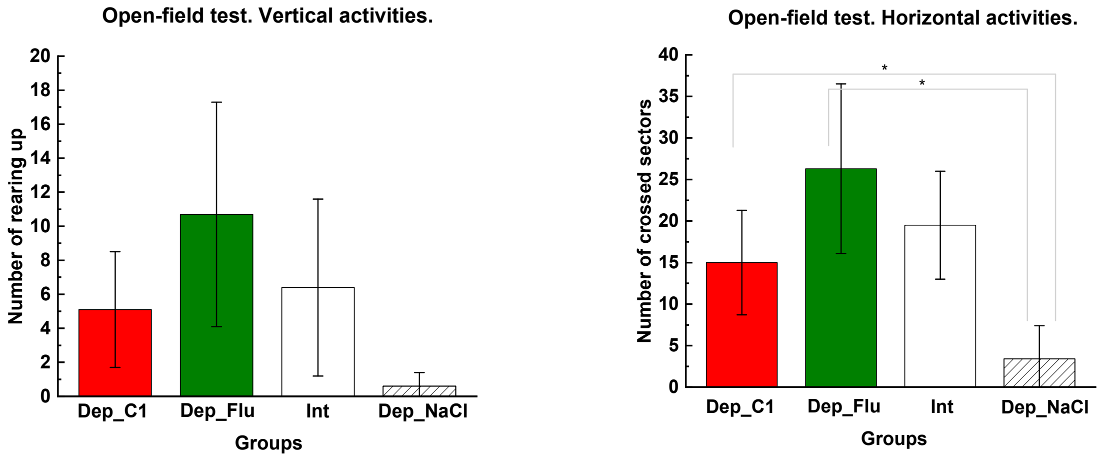

Figure S1: Results of the Open-field test: (**a**) vertical and (**b**) horizontal activities. The results are presented as the mean value ± standard error. Compared to the animals treated with physiological saline, * $p<0.01$ by one-way ANOVA followed by post hoc analysis by Fisher's LSD test

Table S1: Data of HBT conducted on the 0 and 14th days of the experiment

Rat number	Number of crossed sectors		Group after 0d	Group after 14d
	0 day	14 day		
1	25	7	Depression	Compaund I
2	23	5	Depression	Compaund I
3	58		Excluded	
4	31	8	Depression	Fluoxetine
5	42	1	Depression	NaCl 0,9%
6	36	1	Depression	Compaund I
7	35	7	Depression	Compaund I
8	30	7	Depression	Compaund I
9	24	4	Depression	NaCl 0,9%
10	20	2	Depression	Fluoxetine
11	26	1	Depression	NaCl 0,9%
12	23	4	Depression	Compaund I
13	30	13	Intact	Intact
14	22	0	Depression	Compaund I
15	40	3	Depression	Fluoxetine
16	42	7	Depression	Compaund I
17	30	11	Depression	Fluoxetine
18	31	6	Depression	Compaund I
19	41	4	Depression	Compaund I
20	30	7	Depression	Compaund I
21	19	5	Depression	Compaund I
22	22	8	Depression	Fluoxetine
23	39	23	Intact	Intact
24	20	23	Intact	Intact
25	28	18	Intact	Intact

26	20	2	Depression	NaCl 0,9%
27	29	8	Depression	NaCl 0,9%
28	43	1	Depression	Fluoxetine
29	30	10	Depression	NaCl 0,9%
30	20	17	Depression	**No depression**
31	25	32	Intact	Intact
32	24	22	Intact	Intact
33	32	3	Depression	Fluoxetine
34	8		Excluded	
35	29	10	Depression	Fluoxetine
36	35	8	Depression	NaCl 0,9%
37	29	37	Intact	Intact
38	37	21	Intact	Intact
39	38	28	Depression	**No depression**
40	21	8	Depression	NaCl 0,9%
41	33	2	Depression	NaCl 0,9%
42	26	5	Depression	Fluoxetine
43	18	37	Intact	Intact
44	25	21	Intact	Intact
45	34	10	Depression	Fluoxetine
46	17	2	Depression	Fluoxetine
47	26	4	Depression	Fluoxetine
48	28	7	Depression	NaCl 0,9%
49	26	3	Depression	NaCl 0,9%
50	21	6	Depression	NaCl 0,9%
51	43	19	Intact	Intact
52	26	28	Intact	Intact

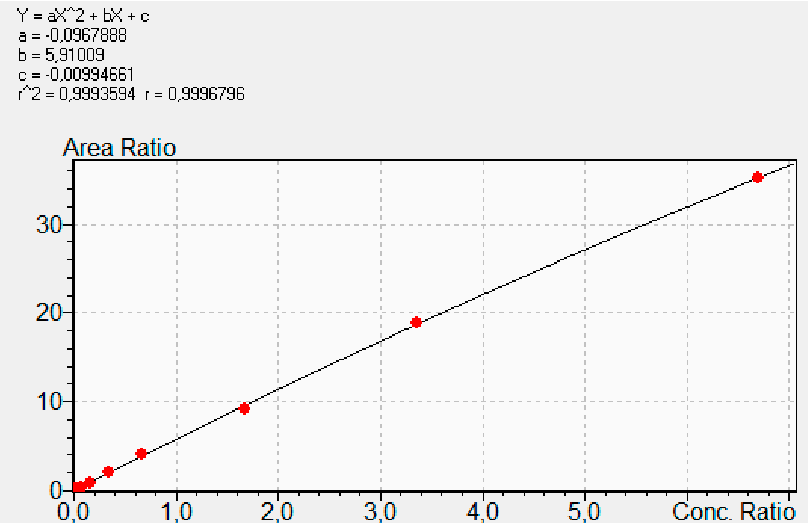

Figure S2. Calibration curve for dopamine determination. Internal standard method. Quadratic dependence, weight coefficient 1/C.

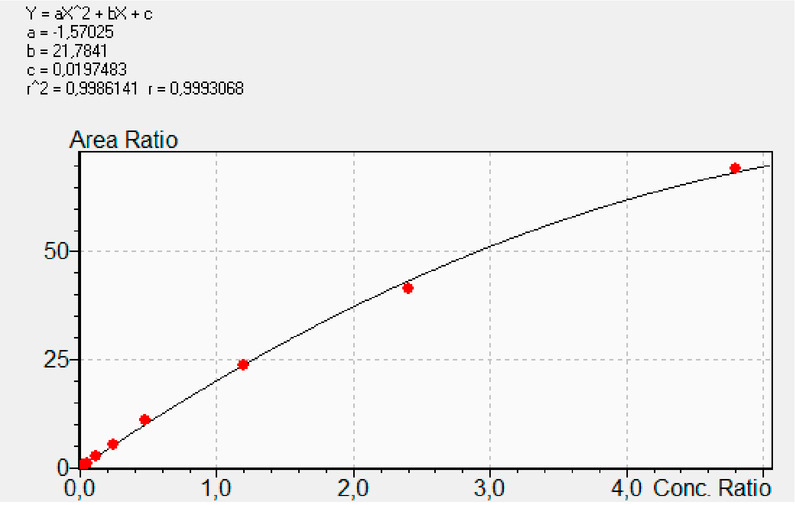

Figure S3. Calibration curve for serotonin determination. Internal standard method. Quadratic dependence, weight coefficient 1/C.

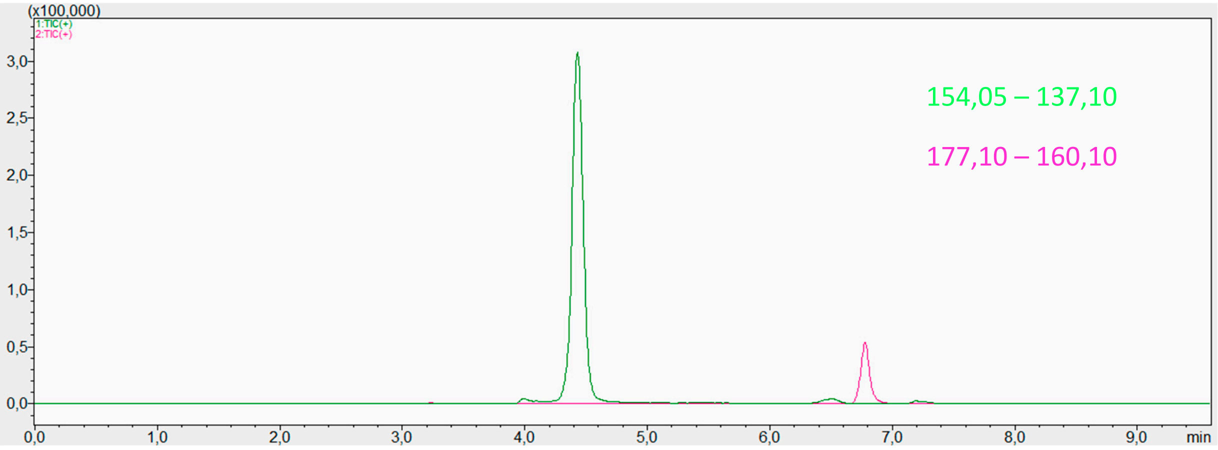

Figure S4. Striatum multiple reaction monitoring (MRM) chromatogram. Positive ion mode (ESI+). Dopamine green, serotonin – pink, Figure S5 Striatum multiple reaction monitoring (MRM) chromatogram. Negative ion mode (ESI–). Caffeic acid (internal standart)

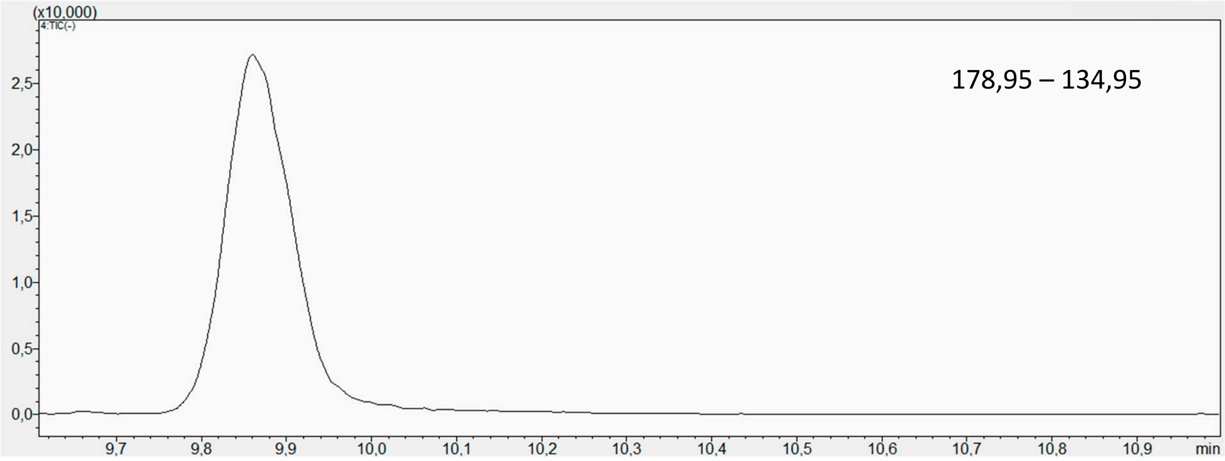

Figure S5. Striatum multiple reaction monitoring (MRM) chromatogram. Negative ion mode (ESI–). Caffeic acid (internal standart).

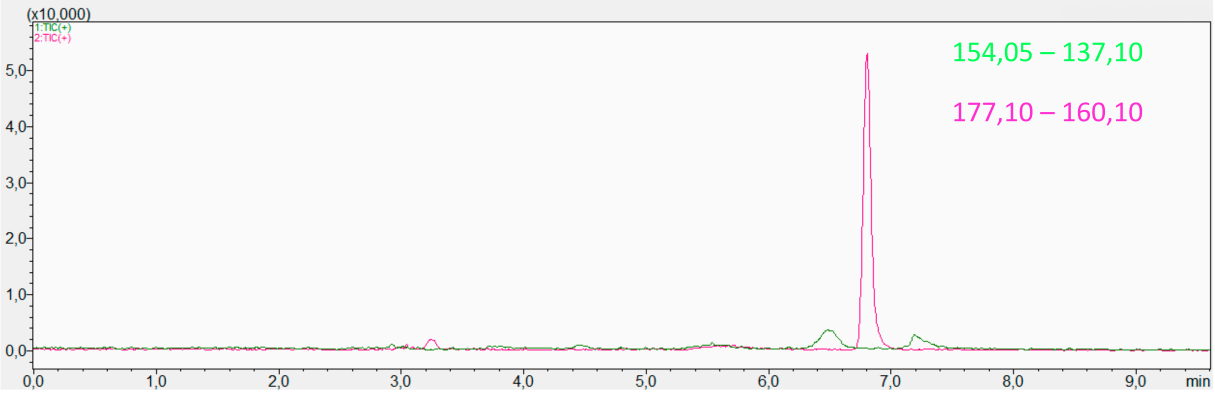

Figure S6. Hippocampus multiple reaction monitoring (MRM) chromatogram. Positive ion mode (ESI+). Dopamine green, serotonin – pink

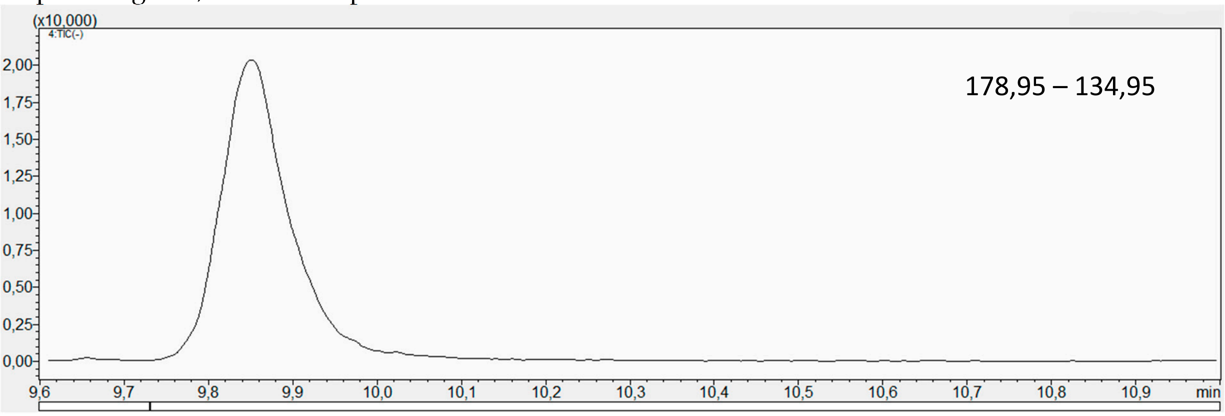

Figure S7. Hippocampus multiple reaction monitoring (MRM) chromatogram. Negative ion mode (ESI–). Caffeic acid (internal standart).
